# Optimized vs. Standard Automated Peritoneal Dialysis Regimens (OptiStAR): study protocol for a randomized controlled crossover trial

**DOI:** 10.1186/s40814-020-00620-2

**Published:** 2020-06-10

**Authors:** Karin Bergling, Javier de Arteaga, Fabián Ledesma, Carl Mikael Öberg

**Affiliations:** 1 Division of Nephrology, Department of Clinical Sciences Lund, Lund University, Alwall House, Skåne University Hospital, Barngatan 2, 22185 Lund, Sweden; 2 Servicio de Nefrología, Hospital Privado Universitario de Córdoba, Universidad Católica de Córdoba, Naciones Unidas 346, 5016 Córdoba, Argentina

**Keywords:** Renal replacement therapy, Automated peritoneal dialysis, Glucose absorption, Metabolic cost

## Abstract

**Background:**

It has been estimated that automated peritoneal dialysis (APD) is currently the fastest growing renal replacement therapy in the world. However, in light of the growing number of diabetic patients on peritoneal dialysis (PD), the unwanted glucose absorption during APD remains problematic. Recent results, using an extended 3-pore model of APD, indicated that large reductions in glucose absorption are possible by using optimized bi-modal treatment regimens, having “UF cycles” using a higher glucose concentration, and “Clearance cycles” using a low concentration or, preferentially, no glucose. The present study is designed to test the theoretical prediction of a lower glucose absorption using these novel regimes.

**Methods:**

This study is a randomized single-center, open-label, prospective study. Prevalent PD patients between 18 and 75 years old without known catheter problems or recent peritonitis are eligible for inclusion. Patients are allocated to a first treatment session of either standard APD (6 × 2 L 1.36% over 9 h) or optimized APD (7 × 2 L 2.27% + 5 × 2 L 0.1% over 8 h). A second treatment session using the other treatment will be performed in a crossover fashion. Samples of the dialysis fluid will be taken before and after the treatment, and the volume of the dialysate before and after the treatment will be carefully assessed. The primary endpoint is difference in glucose absorption between the optimized and standard treatment. Secondary endpoints are ultrafiltration, sodium removal, Kt/V urea, and Kt/V Creatinine. The study will be closed when a total of 20 patients have successfully completed the interventions or terminated according to interim analysis. A Monte Carlo power analysis shows that the study has 80% power to detect a difference of 10 g (in line with that of theoretical results) in glucose absorption between the two treatments in 10 patients.

**Discussion:**

The present study is the first clinical investigation of optimized bi-modal treatments proposed by recent theoretical studies.

**Trial registration:**

ClinicalTrials.gov identifier: NCT04017572. Registration date: July 12, 2019, retrospectively registered.

## Background

In peritoneal dialysis (PD), the peritoneum of the patient is used as a biological dialyzer membrane by filling and draining the peritoneal cavity with dialysis fluid at pre-determined time points. Excess water is removed from the patient by osmosis, which is usually induced by the presence of high concentrations of glucose in the dialysate. The patient absorbs a significant amount of this glucose. In the light of the growing number of diabetic patients on PD, this unwanted glucose absorption is problematic and often represents a clinical challenge. A recent meta-analysis indicated that around 50% of PD patients may develop a glucose disorder which can significantly increase mortality [1].

Automated peritoneal dialysis is PD performed with the aid of a machine, a cycler, which aids the patient or the caregiver from the tedious work of filling and draining the PD fluid. Novel cyclers allow the treatment to be varied in a multitude of ways and recently, it was shown, using a theoretical model, that automated peritoneal dialysis could be improved by using a modified “bi-modal” treatment regimen in which exchanges using no osmotic agent is alternated with short “UF exchanges” having an osmotic agent [2]. Especially, it was shown that the potentially harmful glucose absorption could be reduced by 20–30%. Thus, the potential benefits of the novel regimens appear to be substantial.

The current pilot study aims to evaluate the clinical safety and feasibility of such optimized regimens in a small study using the HomeChoice Pro cycler. Since HomeChoice does not support exchanges having different lengths, the treatment will be divided into a “UF-part” comprising all the UF cycles and a “Clearance part”. A nurse will supervise the treatment and switch between the two parts manually. Moreover, as 0% glucose PD fluid is not available, a 0.1% glucose hemodialysis (CRRT) fluid will be used. We will use 2.27% glucose (Dianeal) for the UF exchanges.

## Method

### Hypothesis

We will test the hypothesis that there is a 10-g difference in terms of glucose absorption, between a standard APD (6 × 2 L 1.36% over 9 h) regime and an optimized APD regimen (7 × 2 L 2.27% + 5 × 2 L 0.1% over ~ 8 h) while there are no differences in osmotic water transport (“UF”), sodium removal, Kt/V creatinine, or Kt/V urea between the regimens.

### Study design

**Fig. 1:**
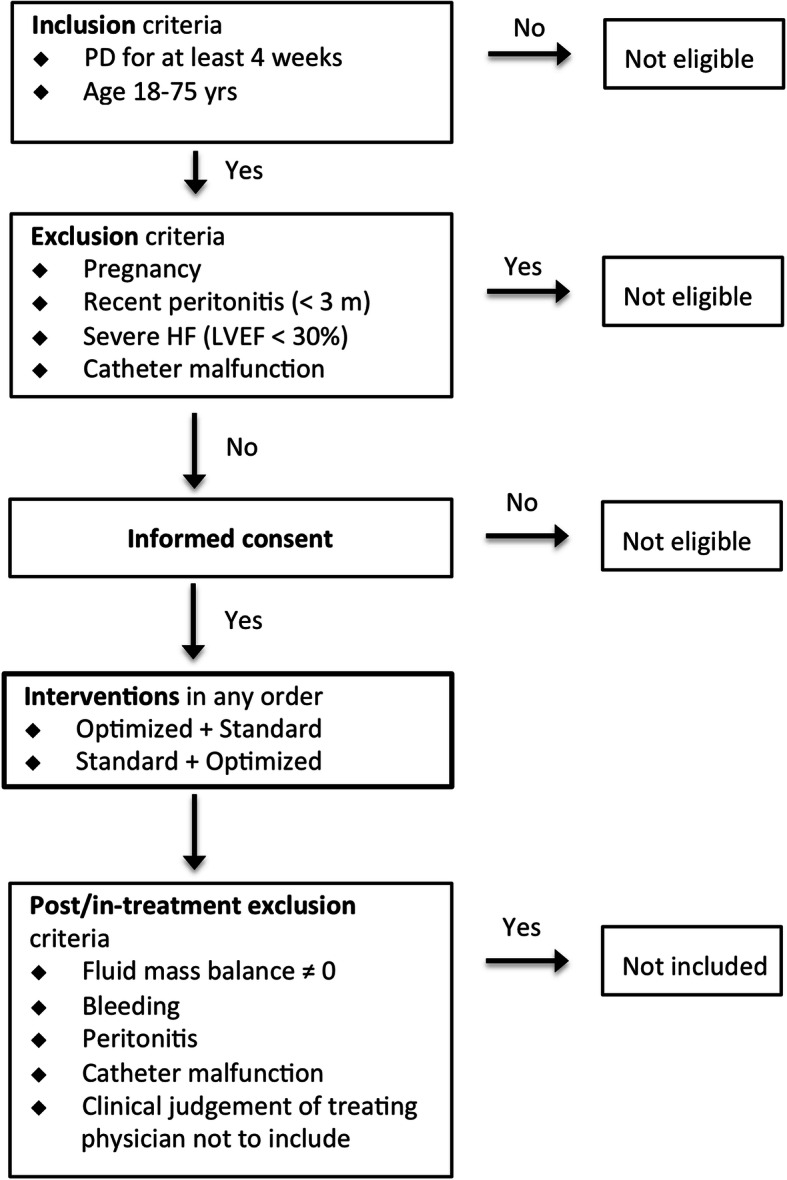
Detailed enrollment and allocation flowchart for the Optimized vs. Standard APD regimens (OptiStAR) study

We will be conducting a randomized single-center, investigator-initiated, prospective, open-label study of two different APD regimens. The study is approved by the regional ethical vetting board in Córdoba, Argentina (Health ministry document registry number 3788), and will be conducted at the Hospital Privado Universitario in Córdoba. Overviews of the trial design according to the Standard Protocol Items: Recommendations for Interventional Trials (SPIRIT) statement and an overview of patient flow through the study are presented in Table 1 and Fig. 1, respectively. A SPIRIT checklist is provided in a supplemental file. A member of the research team will obtain informed consent non-consecutively from eligible participants. The length of the study will be from signing of informed consent until 30 days after the start of the first treatment session. A study investigator will follow all patients in a visit at the trial center. All patients will be evaluated with regard to potential adverse effects by one of the investigators.

**Table 1:** Overview of enrolment, interventions, and assessments according to the SPIRIT (Standard Protocol Items: Recommendations for Interventional Trials) statement

	Study period							
	Recruitment	Allocation	Post-allocation					Close-out
Time point	−t_1_	0	t_1_	t_2_	t_3_	t_4_	t_5_	t_6_
Recruitment								
► Eligibility	**x**							
► Informed consent	**x**							
► Allocation		**x**						
Interventions								
► Optimized APD				**x**	**x**			
► Standard APD				**x**	**x**			
Assessments								
► 4 h D/P creatinine		**x**						
► Urea DV		**x**						
► Glucose absorption				**x**	**x**			
► UF and NaR				**x**	**x**			
► Blood chemistry			**x**	**x**	**x**	**x**		
► Physical examination			**x**	**x**	**x**	**x**	**x**	
► Kt/V urea+crea				**x**	**x**			
► Adverse events				**x**	**x**	**x**	**x**	**x**

*DV* distribution volume, *D/P* dialysate-over-plasma concentration ratio, *UF* net drained volume, *NaR* sodium removal, *Kt/V* calculated from net urea removal divided by plasma urea concentration and DV

### Study participants and pre-intervention assessment

All patients will be selected from the outpatient renal clinic at the Hospital Privado Universitario in Córdoba, Argentina. Participants who provide written informed consent and who meet all of the inclusion and none of the exclusion criteria will be eligible for this study. After allocation, the patient will visit the clinic for screening, and a physical examination will be performed, and a blood samples will be collected. Prior to enrollment, the patient will perform a 4-h dwell using 2.27% glucose, and the dialysate-to-plasma (D/P) concentration ratio of creatinine will be determined. The urea distribution volume (V) will be estimated using the Watson equation.

### Pre-allocation inclusion and exclusion criteria

Inclusion criteria are age between 18 and 75 years, duration of PD (automated peritoneal dialysis (APD), or continuous ambulatory peritoneal dialysis (CAPD)) > 4 weeks.

Exclusion criteria are severe heart failure (New York Heart Association Functional Classification; NYHA III or IV), pregnancy, catheter malfunction, or peritonitis within 3 months prior to the trial.

### Post-allocation exclusion criteria

After allocation, patients may be excluded due to in-trial peritonitis, catheter malfunction, inability to successfully complete both APD regimens, or the clinical judgment of the treating physician not to include the patient.

### Intra- and post-interventional care of the patients

Eligible patients who have given consent to participate in the study will receive routine care. The interventions will start in the morning, and the patient will come to the clinic. After an initial rinse with 1.36% glucose, each patient thereafter receives either a standard or optimized APD in-clinic. After the treatment, a rinse will be performed using 1.36% glucose.

### Randomization and blinding

Consenting patients that fulfill the inclusion criteria and meet no exclusion criteria will be randomized by the investigators using a random number generator to start treatment with either the standard APD or optimized APD regime. A priori we expect no difference in who receives which treatment first. Allocation concealment will be performed using a sealed opaque envelope.

### Study interventions

**Fig. 2:**
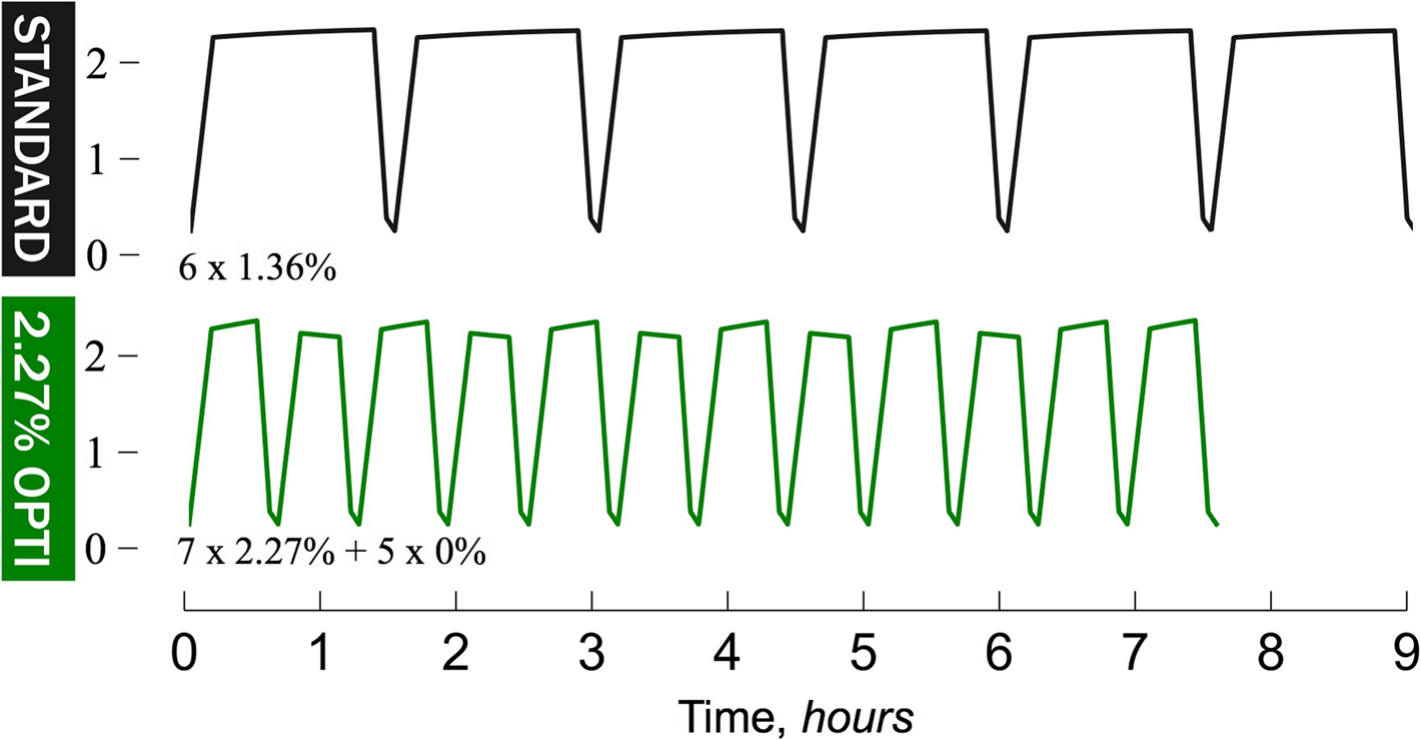
Intra-peritoneal volume as a function of treatment time in hours for **a** a standard 9 h 6 × 2 L 1.36% regime (dwell time 71 min) vs. **b** an modified optimized 7 × 2.27% + 5 × 0% APD regime (dwell time 20 min)

Patients starting with a standard APD regimen will receive treatment with 12 L of dialysis fluid (Dianeal 1.36% glucose) over 9 h (see Fig. 2a). The patient will then, within 4 weeks (1 − 28 days), receive an optimized APD regimen using 7 × 2 L of 2.27% Dianeal during 280 min followed by 5 × 2 L 0.1% glucose fluid (Certesol 0/3.5, Rivero) during 200 min (see Fig. 2b) (lactate-based fluid for continuous hemodialysis/hemodiafiltration). The dwell times for the optimized regimes should theoretically be varied depending on the transport type, but for simplicity, the dwell times have been set fixed in accordance with the above treatment times. This means that the total time under treatment will be approximately 8 h for the optimized regime. Patients will be in the supine position throughout the study protocol session time. Samples of dialysate will be collected from all drained bags (including the initial rinse), immediately after instillation of the first cycle and directly after instillation of the post-treatment rinse dwell.

### Primary outcome

The primary outcome in the study is the amount of glucose (in grams) absorbed from the dialysate during the treatment (glucose absorption).

### Secondary outcomes

Secondary outcomes are osmotic water transport (“UF”), sodium removal, Kt/V urea, Kt/V creatinine, and incidence of complications up to 14 days post-intervention.

### Measurements

The weight of all bags of dialysis fluid, connectors, and drain bags will be carefully recorded before and after treatment start to assess the amount of fluid instilled and removed from the patient. The glucose, urea, creatinine, albumin, total protein, chloride, and sodium concentration of the effluent as well as the fresh dialysis fluid will be measured using the local hospital laboratory*.*

### Data collection and management

All study data will be recorded in case report forms (CRFs) for each patient, which are kept at the study site. Information on co-morbidities, medications, and routine laboratory analysis results will be collected from the hospital electronic chart system. The inclusion criteria will be registered in CRFs before allocation. All members of the research team have unlimited access to study data. The CRFs will be checked continuously during the study period by the study investigators to detect deviations from protocol.

### Sample size and power analysis

Published relative standard error values for glucose absorption comprise values ranging from 10 to 30% with a mean of 18% [3]. The worst performance for the optimized regime is a reduction in glucose absorption of about 10 g. Thus, a Monte Carlo-based power analysis was performed by the generation of 10,000 random samples *S* and *O* of size *N* from two normal sampling distributions having *μ* = 44 g; *σ* = 7.74 and *μ* = 34 g; *σ* = 5.94. The theoretically expected difference will differ depending on transport type and is higher with a faster peritoneal membrane. A Wilcoxon rank-sum test was performed to assess the statistical difference between the samples *S* and *O*. The statistical power was calculated as the number of significant (*P* < 0.05) results, for example, 8687/10,000 implies a statistical power of ~ 87%. The procedure was repeated by step-wise increasing *N* until a statistical power of > 80% was attained, which occurred at *N* = 10. For *N* = 20, the statistical power was found to be > 98%.

### Statistical analysis plan

The study will continue until a total of 20 patients have been included. This number is higher than that estimated in the power analysis. However, only patients who successfully completed both treatment regimens will be included in the analysis. Due to the complicated technical nature of the experimental setup, there may be a significant amount of unusable results, and the relative over-recruitment is aimed at compensating for a high post-allocation exclusion rate. The investigating team will perform the statistical analyses. In general, (1) analysis will be performed on a per-protocol basis, (2) all hypothesis tests will be paired and two-sided, with a maximal type I error risk of 0.05 ,and (3) imputation will not be used to correct for missing data in the analysis.

### Assessment of baseline variables

Baseline variables of all included patients will be tabulated. Discrete variables will be reported as frequencies and percentages, and continuous variables will be reported as either means with SDs or medians with interquartile ranges as appropriate.

### Analysis of outcomes

Study outcomes will be analyzed using a Wilcoxon rank-sum test. A non-parametric test was chosen since such tests are more robust to outliers, which can have undue influence on the results of a parametric test for a small number of patients. In the event of no difference between the groups with regard to secondary outcomes and a difference in the primary outcome (glucose absorption lower in the optimized group), we will interpret such results as supporting our hypothesis but that confirmatory studies are needed using a higher number of patients since the low *N* in the current study may not be adequately powered to detect differences in the secondary outcomes. In this situation, a Monte Carlo-based sensitivity analysis will be performed to quantify the minimal difference needed to yield a significant difference in the secondary outcomes.

In the case of both a significant difference between the groups with regard to secondary outcomes and in the primary outcome (with glucose absorption lower in the optimized group), we will interpret these results as supportive of our hypothesis if the difference in secondary outcomes implies an improvement. Indeed, due to stirring effects, the higher DFR associated with the optimized regimes may result in improved NaR, urea, and creatinine clearance and UF [2]. In any other case, the differences will be compared to the differences expected theoretically according to the extended 3-pore model [2].

Should the primary outcome be negative or show a higher glucose absorption for the optimized regimen, then, all other outcomes are regarded as exploratory, with the exception of number of complications, and no emphasis will be placed on any differences between the treatment groups.

### Good clinical practice and quality assurance

Good clinical practice (GCP) is a well-established, international, ethical, and scientific quality standard for designing, conducting, recording, and reporting clinical trials involving the participation of human subjects. We will comply with GCP to ensure that the rights, safety, and well-being of trial subjects are protected, in agreement with the principles that have their origin in the Declaration of Helsinki, and that the collected data are credible. Quality assurance will be performed by an appointed study coordinator to ensure that the trial is performed and data is recorded and reported in compliance with good clinical practice and the applicable regulatory requirements, and that the study is compliant with the current versions of the Declaration of Helsinki, the International Council for Harmonization of Technical Requirements for Pharmaceuticals for human use, good clinical practice, and national regulations. Considering that healthcare professionals will perform the study using fluids that are certified for either intravenous or intra-peritoneal use, this study will be performed without the use of a data monitoring board. Important protocol amendments will be communicated by the study sponsor to relevant parties.

### Interim analysis

An interim analysis for assessment of efficacy and futility will be performed after 10 patients have completed the protocol. The Haybittle-Peto boundary will be used when testing for efficacy. The study may be stopped if a difference with regard to the primary endpoint of *P* ≤ 0.001 is detected. Futility will be assessed by simulating the remainder of the study multiple times using a standard deviation of 18% and a difference in means of 9 g between the two groups. The results of each simulation will be combined with the obtained data. If the simulated data in combination with the observed data show a significant effect (two-sided Student’s *t* test with an *α* < 0.05) in less than 10% of the cases, the study will be stopped. The principal investigators have the authority to stop the trial.

### Harms

The investigators will evaluate all patients with regard to potential adverse events (AEs) or serious adverse events (SAEs). All potential AEs and SAEs are recorded in the CRF. We define a SAE as an event during the study period that fulfills one or more of the following criteria: results in death, is life-threatening, requires prolongation of hospitalization, results in persistent or significant disability or incapacitation, or any other important medical event. The principal investigators are responsible for the treatment of AEs or SAEs until resolution. Depending on the nature of the AE or SAE, treatment may take place on-site, at the local hospital, or as an outpatient. Principal investigator is responsible for reporting AEs to the institutional review board, participating investigators, and applicable regulatory authorities, Argentine Medical Products Agency (ANMAT), as required per regulations with an expedited copy sent simultaneously to Baxter. All SAEs/significant safety concerns will be sent to Baxter within 24 h. If the principal investigator deems the SAE as being related to the technical equipment or the fluids used, this will be promptly reported to the sponsor, which has the responsibility to report to the Argentine Medical Products Agency (ANMAT) and the local and regional ethical vetting board.

### Publication plan

This study is registered in the ClinicalTrials.gov database (NCT04017572). Following completion of the trial, the manuscript will be submitted to a peer-reviewed journal, regardless of the trial outcome. For publication of the main outcomes, the first figure presented will be a Consolidated Standards of Reporting Trials (CONSORT) flowchart. The diagram will include the number of screened patients, the number of patients giving consent, the number of patients meeting all inclusion criteria, and the number of patients completing the protocol in each of the treatment groups. The second figure will depict glucose absorption for the respective treatments. The first table shall describe baseline variables as described above. The second table will describe secondary outcomes. Authorship will be granted according to the criteria described by the International Committee of Medical Journal Editors.

## Discussion

It is well established that fluid overload is associated with an increased mortality in PD patients [4]. By contrast, small solute clearance seems to have less impact on hard outcomes such as mortality [5, 6]. However, there is an increase in blood sugar levels associated with higher glucose strengths and thus more aggressive fluid management is not unproblematic. Here, the recently suggested optimized APD regimens may be used to improve the treatment considerably. In addition, combined solutions using both icodextrin and glucose have shown a markedly lowered metabolic cost in terms of g glucose absorbed per mL UF [7]. While we expect no difference in sodium removal between the regimens, we do however expect a lower sodium removal in APD with respect to CAPD, mainly due to the shorter dwell times involved. Indeed, a recent meta-analysis including 683 patients showed that CAPD offers a higher sodium removal than APD even though UF is not different [8].

Compared to hemodialysis (HD), PD clearly leads to lower small-solute clearances. Nevertheless, it should be noted that, in terms of symptoms, PD patients might exhibit less uremic symptoms at a higher blood urea nitrogen level compared to HD patients, and thus the concept of using Kt/V urea as a measure of adequacy has been questioned [6]. Thus, it has been hypothesized that PD removes some other, larger, middle-molecular toxin that are commonly cleared less effectively compared to hemodialyzer membranes [6, 9]. Indeed, the downside of the relatively efficient clearance of larger molecules in PD is albumin loss [10].

The main weakness in the current study is the small sample size, which means that there will be insufficient statistical power to detect anything but large differences in urea Kt/V, crea Kt/V, sodium removal, and UF. Hence, while theoretical modeling predicts no large difference in these parameters, this study cannot be used to confirm that finding. On the other hand, it may be argued that small differences in these parameters are more of a theoretical interest and, in practice, of little clinical interest. To further assess this notion, we will, in the case of non-significant outcomes in UF and small-solute transport, perform a sensitivity analysis to estimate the smallest detectable difference in UF between the treatment groups. Moreover, the theoretically expected difference in primary outcome is caused by two separate effects, namely, reduced glucose absorption due to a higher DFR, increasing the average dialysate glucose concentration, and also, the effect of a higher glucose concentration [3, 4] per se. The results in the current study cannot be used to distinguish between these two effects. Another weakness in the study is the simplicity of the power analysis where it was assumed that the sampling distribution is normal. However, since the actual nature of the sampling distribution is unknown, we chose the normal distribution since, according to the central limit theorem, the sampling distribution will tend to the normal distribution with increasing *N*. Lastly, choosing a non-parametric test may inflate the type II error rate [11] if the underlying distribution is truly normal, and there are no outliers. However, biological data typically does not satisfy one or more of these latter criteria and thus the use of parametric methods in medicine relies heavily on large sample sizes since the results of parametric tests are influenced to a higher degree than non-parametric tests both by outliers and deviations from a normal distribution.

### Trial status

Protocol version 1.0 date February 11, 2019. Recruitment began June 18, 2019 and is ongoing, and is expected to be completed in March 31, 2020. No SAEs have been registered to date (January 12, 2019).

## Supplementary information

**Additional file 1.**

**Additional file 2.**

**Additional file 3.**

## Data Availability

All data collected in the present study will be available in anonymized form from the corresponding author on request.
