# Supplementary material for: Optimized vs. Standard Automated Peritoneal Dialysis Regimens (OptiStAR): study protocol for a randomized controlled crossover trial
Source: Pilot Feasibility Stud. 2020 Jun 10;6:81. doi: 10.1186/s40814-020-00620-2 (PMC7285558; doi:10.1186/s40814-020-00620-2)
Supplement: Supplementary file 2 — Additional file 2. [file 40814_2020_620_MOESM2_ESM.docx]

CASE REPORT FORM

OptiStAR

Optimized vs. Standard Automated Peritoneal Dialysis Regimens (OptiStAR) Study

**Exclusion criteria:**

🞏 catheter malfunction

🞏 severe heart failure (LVEF < 30%)

🞏 recent peritonitis (less than 3 mo ago)

🞏 pregnancy

🞏 treating physician’s decision not to include

**Inclusion criteria:**

🞏 informed consent

🞏 age 18-75

🞏 PD for at least 4 weeks

**Background data Date YY-MM-DD ________________**

**PD-start (YY-MM-DD)** _____________

**Length (cm) ________**

**Weight (kg) __________**

**Ideal body weight ______________**

Male: 51 + 0.9 x (length-152.9)

Female: 45.5 + 0.91(length-152.9)

## Date of Birth:

**Year Month Day**

______________________

**Sex:** 🞎 M 🞎 F

**Cerebrovascular disease:**

No 🞎

**Yes 🞎** **○ TIA ○ stroke ○ other: ________________________________________­­­__**

**Lung disease:**

No 🞎

**Yes 🞎** **○ COPD ○ other: ­__________________________**________________________

**Dyspne:**

No 🞎

**Yes 🞎**

| **○** | No Dyspnoea |
| --- | --- |
| **○** | Dyspnoea on exertion |
| **○** | Limiting dyspnoea (on flight), moderate COPD |
| **○** | Dyspnoea at rest (rate > 30/min), lung fibrosis or consolidation |

**Diabetes:**

No 🞎

Yes 🞎 _______________Insulin?_______________

**Heart failure:**

No 🞎

**Yes 🞎** **○** **periph.edema ○ enlarged heart ○ low EF (<40%)**___________________________

**Ischemic heart disease:**

No 🞎

**Yes 🞎** **○ MI<6 months ago ○ MI>6 months ago ○ angina**

**Malignancy:**

No 🞎

**Yes 🞎** ___________________________________________________________________

**Physical exam:**

Blood pressure_______________

Heart frequency____________________

Remarks____________________________________________________________________

####

Drugs

­­­­ Comment

β-blocker No 🞎 Yes 🞎____________________________________

ACE i No 🞎 Yes 🞎____________________________________

AT II antagonist No 🞎 Yes 🞎____________________________________

Statin No 🞎 Yes 🞎____________________________________

Diuretics No 🞎 Yes 🞎____________________________________

ASA No 🞎 Yes 🞎____________________________________

Insulin No 🞎 Yes 🞎____________________________________

Peroral anti-diabetics No 🞎 Yes 🞎____________________________________

Corticosteroids No 🞎 Yes 🞎____________________________________

Inhalations No 🞎 Yes 🞎____________________________________

Warfarin No 🞎 Yes 🞎____________________________________

Nitric oxide No 🞎 Yes 🞎____________________________________

Digoxin No 🞎 Yes 🞎____________________________________

PD modality CAPD 🞎 APD 🞎

4h Dialysate/plasma Crea Date

Pre-intervention lab. Data (Blood sample #1) Date

| Hb Hct | Crea | Calcium | Albumin |
| --- | --- | --- | --- |
| Potassium | CRP | Phosphate |  |
| Glucose | Urea | Sodium |  |

Post-intervention lab. data (Blood sample #2) Date

| Hb Hct | Crea | Calcium | Albumin |
| --- | --- | --- | --- |
| Potassium | CRP | Phosphate |  |
| Glucose | Urea | Sodium |  |

Batch Dialysis Fluid 1.36% Dianeal lab. data Date

| Glucose | Potassium | Albumin |
| --- | --- | --- |
| Sodium | Urea | Total protein |
| Chloride | Crea |  |

Batch Dialysis Fluid 2.27% Dianeal lab. data Date

| Glucose | Potassium | Albumin |
| --- | --- | --- |
| Sodium | Urea | Total protein |
| Chloride | Crea |  |

Batch Dialysis Fluid CRRT fluid lab. data Date

| Glucose | Potassium | Albumin |
| --- | --- | --- |
| Sodium | Urea | Total protein |
| Chloride | Crea |  |

6 x 2L 1.36% TREATMENT PROTOCOL

| STEP | × | Comments |
| --- | --- | --- |
| Priming of Homechoice machine (3 x 6Lts 1.36%) |  |  |
| Physical examination |  |  |
| Blood sample #1 |  |  |
| Drain night bag, discard effluent |  |  |
| Pre-rinse with 1.36% Dianeal |  | Bag weight before fill:  Bag weight after fill: |
| Drain |  |  |
| Dialysate sample #1 10 mL from effluent of 1.36% Dianeal |  |  |
| Connect to HomeChoice and start treatment |  |  |
| Dialysate sample #2 10 mL immediately after first fill |  |  |
| Run treatment (approx 9 hours) |  |  |
| Dialysate sample #3 10 mL during last drain from sample connector on drain line |  |  |
| Disconnect patient from machine |  |  |
| Fill with 1.36% Dianeal |  | Bag weight before fill:  Bag weight after fill: |
| Dialysate sample #4 10 mL immediately after fill |  |  |
| Drain |  |  |
| Dialysate sample #5 10 mL from treatment drain bag |  |  |
| Blood sample #2 |  |  |

| Dialysate sample #1   \| Glucose \| Urea \| Potassium \| \| --- \| --- \| --- \| \| Sodium \| Albumin \| Chloride \| \| Crea \| Total protein \|  \|   Dialysate Sample #2   \| Glucose \| Urea \| Potassium \| \| --- \| --- \| --- \| \| Sodium \| Albumin \| Chloride \| \| Crea \| Total protein \|  \|   Dialysate Sample #3   \| Glucose \| Urea \| Potassium \| \| --- \| --- \| --- \| \| Sodium \| Albumin \| Chloride \| \| Crea \| Total protein \|  \|   Dialysate Sample #4   \| Glucose \| Urea \| Potassium \| \| --- \| --- \| --- \| \| Sodium \| Albumin \| Chloride \| \| Crea \| Total protein \|  \|   Dialysate Sample #5   \| Glucose \| Urea \| Potassium \| \| --- \| --- \| --- \| \| Sodium \| Albumin \| Chloride \| \| Crea \| Total protein \|  \| |
| --- | --- | --- | --- | --- | --- | --- | --- | --- | --- | --- | --- | --- | --- | --- | --- | --- | --- | --- | --- | --- | --- | --- | --- | --- | --- | --- | --- | --- | --- | --- | --- | --- | --- | --- | --- | --- | --- | --- | --- | --- | --- | --- | --- | --- | --- |

Volumes:

| Treatment drain volume: |
| --- |
| UF (measured by machine): |

OPTIMIZED TREATMENT PROTOCOL

| STEP | × | Comments |
| --- | --- | --- |
| Priming of HomeChoice machine #1 (3 x 6Lts 2.27%) |  |  |
| Physical examination |  |  |
| Blood sample #1 |  |  |
| Drain night bag, discard effluent |  |  |
| Pre-rinse with 1.36% Dianeal |  | Bag weight before fill:  Bag weight after fill: |
| Dialysate sample #1 10 mL from effluent of 1.36% Dianeal |  |  |
| Drain |  |  |
| Connect to HomeChoice #1 and start treatment (2.3%) |  |  |
| Dialysate sample #2 10 mL immediately after first fill |  |  |
| Run treatment (approx. 280 min/4h 40 min) |  |  |
| Priming of HomeChoice machine #2 (4 x 3Lts 0.1%) |  |  |
| Dialysate Sample #3 10 mL during last drain from sample connector on drain line |  |  |
| Disconnect patient from machine #1 |  |  |
| Dialysate sample #4 10 mL from treatment drain bag #1 (2.3%) |  | Weight: |
| Weight of treatment drain bag #1 (2.3%) |  |  |
| Connect to HomeChoice #2 and start treatment (0.1% glucose) |  |  |
| Dialysate sample #5 10 mL immediately after first fill |  |  |
| Run treatment (approx. 200 min/3h 20 min) |  |  |
| Dialysate sample #6 10 mL during last drain from sample connector on drain line |  |  |
| Disconnect patient from machine #2 |  |  |
| Fill with 1.36% Dianeal |  | Bag weight before fill:  Bag weight after fill: |
| Dialysate sample #7 10 mL immediately after fill |  |  |
| Drain |  |  |
| Dialysate sample #8 10 mL from treatment drain bag #2 (0.1%) |  | Weight: |
| Weight of drain bag #2 (0.1%) |  |  |
| Blood sample #2 |  |  |

| Dialysate sample #1   \| Glucose \| Urea \| Potassium \| \| --- \| --- \| --- \| \| Sodium \| Albumin \| Chloride \| \| Crea \| Total protein \|  \|   Dialysate Sample #2   \| Glucose \| Urea \| Potassium \| \| --- \| --- \| --- \| \| Sodium \| Albumin \| Chloride \| \| Crea \| Total protein \|  \|   Dialysate sample #3   \| Glucose \| Urea \| Potassium \| \| --- \| --- \| --- \| \| Sodium \| Albumin \| Chloride \| \| Crea \| Total protein \|  \|   Dialysate sample #4   \| Glucose \| Urea \| Potassium \| \| --- \| --- \| --- \| \| Sodium \| Albumin \| Chloride \| \| Crea \| Total protein \|  \|   Dialysate sample #5   \| Glucose \| Urea \| Potassium \| \| --- \| --- \| --- \| \| Sodium \| Albumin \| Chloride \| \| Crea \| Total protein \|  \|   Dialysate sample #6   \| Glucose \| Urea \| Potassium \| \| --- \| --- \| --- \| \| Sodium \| Albumin \| Chloride \| \| Crea \| Total protein \|  \|   Dialysate sample #7   \| Glucose \| Urea \| Potassium \| \| --- \| --- \| --- \| \| Sodium \| Albumin \| Chloride \| \| Crea \| Total protein \|  \|   Dialysate sample #8   \| Glucose \| Urea \| Potassium \| \| --- \| --- \| --- \| \| Sodium \| Albumin \| Chloride \| \| Crea \| Total protein \|  \| |
| --- | --- | --- | --- | --- | --- | --- | --- | --- | --- | --- | --- | --- | --- | --- | --- | --- | --- | --- | --- | --- | --- | --- | --- | --- | --- | --- | --- | --- | --- | --- | --- | --- | --- | --- | --- | --- | --- | --- | --- | --- | --- | --- | --- | --- | --- | --- | --- | --- | --- | --- | --- | --- | --- | --- | --- | --- | --- | --- | --- | --- | --- | --- | --- | --- | --- | --- | --- | --- | --- | --- | --- | --- |

Volumes:

| 2.3% Treatment drain volume: |
| --- |
| 2.3% UF (measured by machine): |
| 0.1% Treatment drain volume: |
| 0.1% UF (measured by machine): |

Adverse Events

Date ______

Start: kl._____

Stop: kl._____

**Description of the event**:_________________________________________________________

______________________________________________________________________________
______________________________________________________________________________

**Action/treatment**_______________________________________________________________

(no action, temporary stopped, permanently stopped)

**Outcome**______________________________________________________________________
(recovered, recovered with sequele, ongoing)

**Severity** (see page 13) mild 🞏 moderate 🞏 severe 🞏

**Causality in relation to the study intervention:** likely🞏 possible🞏 unlikely🞏

If severe, contact Javier de Arteaga (+54 9 351 510-4067) or Carl M Öberg (+46 709 221947) immediately.

Adverse Events

**B** Date ______

Start: kl._____

Stop: kl._____

**Description of the event**:_________________________________________________________

______________________________________________________________________________
______________________________________________________________________________

**Action/treatment**_______________________________________________________________

(no action, temporary stopped, permanently stopped)

**Outcome**______________________________________________________________________
(recovered, recovered with sequele, ongoing)

**Severity** (see page 13) mild 🞏 moderate 🞏 severe 🞏

**Causality in relation to the study intervention:** likely🞏 possible🞏 unlikely🞏

If severe, contact Javier de Arteaga (+54 9 351 510-4067) or Carl M Öberg (+46 709 221947) immediately.

Adverse Events

**B** Date ______

Start: kl._____

Stop: kl._____

**Description of the event**:_________________________________________________________

______________________________________________________________________________
______________________________________________________________________________

**Action/treatment**_______________________________________________________________

(no action, temporary stopped, permanently stopped)

**Outcome**______________________________________________________________________
(recovered, recovered with sequele, ongoing)

**Severity** (see page 13) mild 🞏 moderate 🞏 severe 🞏

**Causality in relation to the study intervention:** likely🞏 possible🞏 unlikely🞏

If severe, contact Javier de Arteaga (+54 9 351 510-4067) or Carl M Öberg (+46 709 221947) immediately.

In case of more than 3 AE, print an extra page 12.

Study Close-Out

Fill this section out when all procedures and blood samples have been performed.

Date : _ _ _ _-_ _-_ _

Year MM DD

→ Completion of the study protocol? Yes □ No □

If not – provide reason:

□ Consent retracted

Reason:_________________________________

□ Lost to follow up

□ Death. Report to Javier de Arteaga within 24h

Deceased date: _ _ _ _ - _ _ - _ _

YY MM DD

Cause of death:________________________________________

Autopsy: no □ yes□ if yes attach report

Principal Investigator

I hereby confirm that the information in this CRF is in correct.

Principal investigator signature:

Name :

Date: _ _ _ _ - _ _ - _ _

YY MM DD

**Definition of Adverse Events**

An adverse event (AE) is any untoward medical occurrence in a patient given a

pharmaceutical product, not necessarily having any causal relationship with the

study treatment. An adverse event can therefore be any unfavourable and unintended sign

(e.g., tachycardia, enlarged liver) or abnormal results of an investigation (e.g., laboratory

finding, ECG), or symptom (e.g., nausea, chest pain) or disease temporally associated with the use of a medical (investigation product), whether or not related to the medicinal

(investigational) product.

**Definition Serious Adverse Events**

A serious adverse event (SAE) is an AE occurring during any part of the study that fulfils one or more of the following criteria:

- results in death,
- is life-threatening,
- requires hospitalisation or prolongation of existing inpatients’ hospitalisation,
- results in persistent or significant disability or incapacity
- other important medical events

All SAEs must be reported to the sponsor within 24 hours after the investigator has

become aware of it.

*Assessment of severity*

All adverse events will be assed for severity by the investigator.

• Mild: Awareness of a clinical sign or a symptom, but easily tolerated and cause no interference with daily activities.

• Moderate: Discomfort enough to cause interference with daily activities.

• Severe: Incapacitating with inability to perform normal daily activities.

*Causal relationship to study intervention*

The investigator will judge whether or not, in his/her opinion; the adverse event is associated with the study treatment.

Probably: An adverse event, which might be due to the use of the treatment. The relationship in time is suggestive. An alternative explanation is less likely, e.g. concomitant treatment(s), concomitant disease(s).

Possibly: An adverse event, which might be due to the treatment/intervention. An alternative explanation, e.g. concomitant drug(s), concomitant disease(s), is inconclusive. The relationship in time is reasonable and, therefore, the causal relationship cannot be

excluded.

Unlikely: An adverse event for which an alternative explanation is more likely, e.g.

other concomitant treatment(s), other concomitant disease(s), or the relationship in time suggests that a causal relationship is unlikely.

**Adverse Event reporting**

During the PD-treatment all patients may experience AEs as defined above. Based

on this, any expected effects will not be reported. Thus, signs and symptoms, which are associated with the normal PD-treatment will therefore only be reported as AEs if it is both:

- Serious according to the above definition and
- Is not an expected symtom or sign as determined by the investigator.

The following variables will be recorded in the CRF for each AE; description, start and stop

date, severity, SAE or not, causality rating, action taken and outcome of the AE.

**Recording of AE**

Adverse events occurring from signing of the consent until end of study will be recorded in the CRF.

**Suspected Unexpected Serious Adverse Reaction (SUSAR)**

All serious adverse events (SAE) must be evaluated unexpected or treatment related or not. The definition of an unexpected adverse reaction is any adverse event, which has not been documented or reported earlier. If the responsible investigator/sponsor judges the SAE as being related to the study treatment and unexpected it must be promptly reported to the sponsor which has the responsibility to report according to local SUSAR regulations.
