# Supplementary material for: Optimized vs. Standard Automated Peritoneal Dialysis Regimens (OptiStAR): study protocol for a randomized controlled crossover trial
Source: Pilot Feasibility Stud. 2020 Jun 10;6:81. doi: 10.1186/s40814-020-00620-2 (PMC7285558; doi:10.1186/s40814-020-00620-2)
Supplement: Supplementary file 3 — Additional file 3. [file 40814_2020_620_MOESM3_ESM.docx]

**Patient information**

OptiStAR Study

**Optimized *versus* Standard APD Regimes**

You are hereby asked to participate in a clinical study of a new optimized treatment with automated peritoneal dialysis. Participation in the study is voluntary and you will receive the same care regardless if you choose to participate or not. At the Hospital Privado we aim to improve the medical treatment and care of all patients by performing clinical research. This study is performed by the clinic in collaboration with Lund University in Sweden. Herein you will find information about how the OptiStAR study will be performed and why. You will also find information on how to gain access to the information that we have gathered. Please, take your time to read this information. Should you have any questions, don’t hesitate to ask us.

**Background and Purpose**

Peritoneal dialysis (PD) is performed by filling and draining the peritoneal cavity with dialysis fluid at pre-determined time points. Excess water is removed from the patient by a phenomenon called osmosis, in which water is drawn to the dialysate due to the presence of high concentrations of glucose in the dialysis fluid. Unfortunately, the patient absorbs a significant amount of this glucose. In the light of the growing number of diabetic patients on PD, this unwanted glucose absorption is problematic and often represents a clinical challenge.

Automated peritoneal dialysis (APD) is PD performed with the aid of a machine, for example the HomeChoice Pro cycler. Recently it was shown, using a theoretical model, that automated peritoneal dialysis could be improved by using so called Optimized APD prescriptions. Especially it was shown that the potentially harmful glucose absorption could be reduced by 20-30% while shortening the treatment time.

In the current study, we aim to evaluate the clinical safety and feasibility of such optimized treatments.

**How will the study be performed?**

The local and regional ethics committees have approved this study. If you chose to participate you will be called to the clinic on 2 occasions to receive treatment with APD. The treatment time will be 8-9 hours. After the first fill, a sample of dialysis fluid will be collected from your PD-catheter. Before and after the treatment, we will perform a rinse with 1.36% glucose. After filling the last rinse, a sample of dialysis fluid will be collected from your PD-catheter. The last rinse will also contain a small amount of antibiotics to prevent infection. About 1 month after the last treatment session, we will call you for a follow-up visit to the clinic. Thereafter your participation in the study is terminated.

**Benefits of participation**

You will have no personal gain from participating in this study. For all participants, blood and dialysate samples will be carefully studied to assess the possible benefits of Optimized APD. The results of the study can lead to improved PD-treatment for other patients in the future.

**Drawbacks of participation**

The days, on which you receive the study treatments, you will not be able to have your regular PD-treatment. We will, on one occasion, use dialysis fluid that is normally used only for hemodialysis. While we do not expect any side effects from this fluid, it has not previously been used for PD. Healthcare professionals will monitor you during the entire treatment.

**Economic reimbursements**

You will not receive any economic compensation for participating in this study. None of the researchers have any financial interests in this study.

**Number of patients**

A total of 20 patients will be included in this study.

**Handling of study data**

All information collected during this study will be confidential and only authorized personnel will have access to your information. Data will be stored in a digital database. I the database your name will be replaced by a unique number so that the data cannot be linked to an individual person. Only the principal investigators will have access to the identity corresponding to each unique number. The study results will be reported in a scientific journal, but only on a group level and no individual information will be revealed.

**Handling of samples**

Samples of blood and dialysate will be analyzed at a clinical chemical laboratory. Before and after analysis the samples will be stored until analysis results have been obtained. The samples will only be used for the purposes stated herein and can only be used for another purpose after approval from the regional ethics board. You have the right to ask that your samples be destroyed without any further explanation.

**Participation is voluntary**

Participation in this study is entirely voluntary and you have the right to cancel your participation at any time without any further explanation. Should there be any new information that may affect your will to participate, you will receive this information. Your treating physician has the right to cancel your participation at any time for medical reasons.

**Contact**

Dr Javier de Arteaga +54 9 351 510-4067.

Dr Carl M Oberg +46 709 22 19 47.

**Patient Consent**

I have read the above information, received answers to my questions and I consent to participation in this study.

I am aware that participation is entirely voluntary.

I am aware that the collected samples may be used for another purpose that stated in the above information, but only if this has been approved by the regional ethics board.

|  |  |  |
| --- | --- | --- |
| Date (YYYY-MM-DD) | | Patient signature |
|  | |  |
|  | | Patient name |

**Treating physician**

I have provided this patient with information about the study. I have answered all questions about the study and ensured that the patient has understood the answers. Thereafter I have obtained written consent. A copy of the written information has been given to the patient.

|  |  |  |
| --- | --- | --- |
| Date (YYYY-MM-DD) | | Treating physician signature |
|  |  |  |
| Location | | Name |
